# Determinants and group differences in international undergraduates’ engagement in Chinese higher education

**DOI:** 10.1371/journal.pone.0337038

**Published:** 2025-11-14

**Authors:** Jun Qu, Dharm Dev Bhatta

**Affiliations:** 1 College of Foreign Languages and International Business, Guilin University of Aerospace Technology, Guilin, Guangxi, China; 2 Yunnan Chinese Language and Culture College, Yunnan Normal University, Kunming, China; Universiti Sains Malaysia, MALAYSIA

## Abstract

Student engagement is a crucial indicator of academic success, quality education, and overall development, encompassing behavioral, cognitive, and emotional dimensions. This study aimed to investigate the levels and key determinants of engagement among international undergraduate students in China, to gain a deeper understanding of the individual and contextual factors that shape their learning experiences. Data were collected from 721 international students enrolled in 21 non-double-first-class universities in Yunnan and Guangxi provinces, using a structured questionnaire adapted from the National Survey of Student Engagement-China, the China College Student Survey, and the Motivated Strategies for Learning Questionnaire. The results revealed a moderate level of overall engagement (M = 4.02 on a 5-point scale), with emotional engagement scoring the highest (M = 4.10), followed by behavioral (M = 3.99) and cognitive engagement (M = 3.98). A gender-based analysis revealed significant differences in cognitive performance (p < 0.01, Cohen’s d = 0.27) and overall engagement (p < 0.05, Cohen’s d = 0.17), with males scoring higher. Academic discipline and grade level were also significantly associated with engagement dimensions, while Chinese language proficiency showed no significant effect. Regression and Structural Equation Modeling(SEM) analyses identified cultural adaptation (β = 0.35), self-efficacy (β = 0.34), teacher behavioral support (β = 0.29), and peer behavioral support (β = 0.25) as strong and significant predictors, collectively explaining over 60% of the variance in students’ overall engagement(χ²/df = 2.12, CFI = 0.98, TLI = 0.97, RMSEA = 0.04, and SRMR = 0.03). The findings suggest that institutions must strengthen culturally responsive pedagogies, enhance support structures, and promote active learning strategies to improve international students’ engagement and academic success in Chinese higher education.

## 1 Introduction

Student engagement (SE) is a multifaceted construct encompassing interrelated and mutually supportive behavioral, emotional, and cognitive dimensions in both academic and co-curricular activities [1]. As a significant predictor of academic performance and personal development, SE reflects the time, energy, and psychological commitment students invest in learning, shaping both individual success and institutional effectiveness [2]. Definitions of SE vary across contexts, but a widely accepted perspective views it as the time and energy students devote to educationally purposeful activities [3,4]. Zepke [5] and Zapke and Leach [1] emphasize SE as what students do, think, and feel when learning, shaped by instructional strategies and institutional environments.

Empirical research has identified SE as a mediator of quality education and learner outcomes [6,7]. It incorporates not only students’ intrinsic attributes, such as participation, motivation, effort, and cognitive engagement, but also external influences, including teacher support, peer interaction, and institutional climate [6,7]. Consequently, SE has become a key construct in shaping educational policy, teaching practices, and curriculum reforms aimed at enhancing student learning experiences across global higher education systems [8].

In recent years, China has emerged as one of the top destinations for international students. By 2021, the number of international students (IS) in China exceeded 700,000, with growth primarily driven by government-sponsored initiatives and Chinese language immersion programs [9]. To standardize academic quality, the Ministry of Education launched the “Quality Standards for Higher Education for International Students in China (Trial)” in 2018, encouraging student-centered, output-oriented, and continuously improving education practices [10]. However, existing literature suggests that ISs in Chinese universities often perceive themselves as less respected and welcomed than their domestic counterparts on campus [11,12]. They usually struggle with academic and cultural integration, limited classroom participation, language barriers, and inadequate peer interaction [12–14]. These challenges are further compounded by the dominant use of Chinese as the medium of instruction and the limited implementation of active learning strategies [15].

Prior studies have explored the engagement of ISs from Europe, the Americas, and East Asian countries enrolled in science and engineering disciplines at elite universities (also known as double-first-class universities), particularly in developed regions of China [13,14,16–18]. However, limited empirical evidence is available on their experiences in under-researched provinces and universities (non-double-first-class institutions), such as in Yunnan and Guangxi. Moreover, few studies have critically examined how specific individual and contextual factors (e.g., cultural adaptation, language proficiency, or teacher and peer support) shape the different dimensions of engagement.

To address this gap, the current study adopts a multidimensional framework of SE, comprising behavioral, cognitive, and emotional engagement. It examines both individual-level (e.g., self-efficacy, motivation) and contextual-level (e.g., teacher/peer support) determinants of SE among international undergraduates in Chinese higher education. By identifying the extent and nature of their engagement, as well as the key factors influencing it, this study aims to inform institutional strategies that promote more inclusive and supportive learning environments for ISs.

This study addresses the following research questions:

RQ1: To what extent do international undergraduates exhibit engagement in Chinese universities across Yunnan and Guangxi?

RQ2: What are the group differences in their engagement level?

RQ3: What variables have the most impact on international undergraduates’ engagement?

## 2 Literature review

### 2.1 Theoretical perspectives on student engagement

In recent years, student engagement has emerged as a crucial construct in higher education research, owing to its significant impact on academic success, personal development, and retention. Defined broadly as the intensity of students’ involvement, interest, and commitment to academic and co-curricular activities [1,19], SE has been operationalized through various frameworks. The conceptual foundation of this study primarily draws from Astin’s Student Involvement (SI) Theory [19] and Kuh’s Learning Engagement (LE) Theory [3]. Astin posits that student engagement is directly related to the quantity and quality of student involvement in academic and social activities, as well as frequent contact with faculty members and peers within their educational setting. While this perspective focuses on the physical (regular attendance, participation in extra-curricular activities) and psychological investment (e.g., motivation, effort) as core drivers of SE and academic pursuits [19]; Kuh, on the other hand, expands this focus to contextual and multidimensional layers, emphasizing behavioral, psychological, socio-cultural, and holistic dimensions that foster learning, proposing that SE depends not only on learners’ effort but also on how institutions structure and support learning opportunities [20,21].

Building on these frameworks, global national surveys, including the National Survey of Student Engagement (NSSE, U.S.), the National Student Survey (NSS-UK), and the Chinese University Student Learning Engagement Survey (NSSE-China), have been operationalized to target different areas and aspects, assessing higher education quality through student-centered metrics [22,23]. While these surveys differ in structure, they commonly address core domains, including academic challenge, active learning, student-faculty interaction, and a supportive learning environment. For instance, NSS-U.K. tracks seven areas, including teaching quality, organizational management, assessment, and feedback. Scholars like Trowler [7] argue that NSS-U.K. is actually less likely to focus on Students’ overall engagement, but rather is overwhelmingly focused on particular aspects of individual student learning, tools, techniques, approaches, and specific situations, such as online learning. In contrast, NSSE focuses on key benchmarks, including the level of academic challenge, active and collaborative learning, student-faculty interaction, and a supportive campus environment, within behavioral, cognitive, emotional, and socio-cultural dimensions [23].

NSSE-China (2007 version), an adaptation of the NSSE-U.S., retains Kuh’s five key indicators (academic challenge, active and collaborative learning, student-faculty interaction, enriching educational experiences, and a supportive campus environment), ensuring cross-context comparability while accommodating localized academic norms [24]. The NSSE-China has been further refined and popularized as the China College Student Survey (CCSS) by modifying items from NSSE-China to measure three core SE dimensions, as outlined in [25], and associated influencing factors [24]. However, few studies have adapted these tools to measure the engagement dimensions of ISs and their predictors in the Chinese context.

Student engagement is typically described as having two to four dimensions/components in various settings. While two-dimensional models often include behavioral (e.g., participation, effort) and emotional or affective (e.g., enthusiasm, belonging) aspects, three-dimensional models also encompass cognitive (e.g., learning goals, self-regulation) aspects. The four-dimensional model integrates social/agentic aspects, including behavioural, emotional, and cognitive aspects [9].

The study combines Kuh’s LE framework with Frederick et al.’s three-dimensional model. It utilizes SE questions from the NSSE-China, CCSS, the Motivated Strategies for Learning Questionnaire (MSLQ) [26], and other validated scales that aim to capture the engagement of international undergraduates over three core dimensions and the contributing factors in Chinese universities in Yunnan and Guangxi.

### 2.2 Dimensions and predictors of student engagement

Student engagement is widely acknowledged as a multidimensional construct comprising behavioral, emotional, cognitive, and social/agentic interrelated domains. Behavioral engagement refers to students’ behavioral responses, such as attending classes, participating in academic tasks, completing assignments, and engaging in discussions (e.g., asking questions, responding to teachers) [27,28]. Emotional engagement encompasses students’ affective responses, including interest, enthusiasm, and a sense of belonging within the school environment [28,29]. Cognitive engagement reflects mental effort through focused attention, the deployment of deep learning strategies (e.g., elaboration and synthesis), and self-regulatory processes [30,31]. Similarly, social/agentic engagement captures proactive initiatives in which learners intentionally influence the instructions, ranging from not knowing, not understanding, and not achieving to knowing, understanding, and achieving [5,32]. The interconnection of these dimensions is essential for promoting deeper learning. For instance, in a virtual discussion, behavioral engagement may be demonstrated by a student composing a post, cognitive engagement through the depth of their ideas, and emotional engagement by their enthusiasm for the subject matter [3,4].

Empirical studies have consistently demonstrated a robust correlation between these dimensions and critical academic outcomes. Students who are behaviourally engaged typically comply with behavioural norms, such as actively participating in classroom discussions and paying attention to instructional materials and directives [4]. This not only boosts motivation and helps them identify opportunities, challenges, and absorb new information, but also aids in developing academic skills and agency [27,28]. In contrast, disruptive behaviors lead to disengagement [33]. Similarly, emotional engagement significantly mediates persistence. Students who engage emotionally experience affective reactions, such as interest and enjoyment, which sustain expectations, facilitate articulation of assumptions, and foster a commitment to learning [29,30]. In contrast, strong negative emotions (e.g., boredom and disappointment) diminish motivation, academic effort, and performance [33]. Cognitively engaged students would be invested in their learning, seek to go beyond the requirements, enable a deep understanding of the discipline, facilitate deep information processing, promote self-directed learning, and enhance application [4,30]. Students who perceive task relevance and possess self-efficacy tend to engage more deeply, thereby enhancing critical thinking and facilitating self-regulated learning [34,35]. In addition to being involved in behavioral, emotional, and cognitive dimensions, agentically engaged students take action before a learning activity begins [32]. Students’ social engagement with peers, teachers, and the broader learning community further predicts resilience, creating a sense of belonging and establishing trust, especially when students proactively navigate barriers [30,34]. Students with positive social interactions, for example, who tell the teacher what they like and dislike, and who offer suggestions on how to improve the class, feel supported in their learning and are more likely to be engaged, and vice versa [32]. Collectively, these dimensions have a direct influence on student persistence, satisfaction, and skill mastery across diverse contexts. Therefore, institutions and teachers should implement programs supporting students’ social and emotional development (e.g., culturally responsive teaching and recognizing and leveraging students’ diverse experiences), which can improve classroom behavior, effort, and participation levels, making learning more relevant and meaningful [36].

Several individual, cultural, and contextual factors moderate students’ engagement dynamics. Individual psychological variables, such as motivation and self-efficacy, are the primary factors that encourage students to participate actively in academic activities and fortify their resilience in the face of academic challenges [37–39]. Both intrinsic and extrinsically motivated students are more resilient in learning problems; however, studies suggest that intrinsic motivation remains more beneficial in supporting students’ need for autonomy as well as overall competence and performance [20]. Similarly, gender and grades may impact students’ overall engagement, academic motivation, and school engagement. Studies by Hassim and Yang [37] and Letaert et al. [38] reveal that overall engagement was higher for males and in higher grades.

Additionally, socio-cultural factors, such as cultural adaptation and language proficiency, shape ISs’ navigation of new academic norms [29–32]. Students are cognitively engaged when they enjoy foreign languages, identify with, and understand school culture [39]. A healthy campus, characterized by interactive student-institutional, student-teacher, and peer relations and support, fosters students’ connectedness and emotional security, which in turn bolsters SE [39–43]. Conversely, lower levels of college, teacher, and peer acceptance, as well as feelings of disconnectedness, are associated with reduced engagement [44,45]. Therefore, it is essential to investigate how individual factors and behavioral and emotional support from institutions, teachers, and peers affect ISs’ engagement and academic performance.

Furthermore, developing 21st-century skills, such as critical thinking, problem-solving, acquire information and engage deeply with content, requires learners to apply technological interventions such as digital tools, AI-driven communication platforms, including chatbots, two-way texting, and live chat [46] These can offer instantaneous, real-time, interactive, personalized learning experiences and tailored information that access to various resources and contributing to a more profound understanding and skill mastery [47].

### 2.3 International student engagement in China

Empirical research on international students’ engagement in Western contexts has extensively examined core SE dimensions, consistently affirming that cultural adaptation, interactive teacher–student dynamics, peer collaboration, and technological interventions in teaching enhance SE [48–50]. Studies on ISs in Chinese universities have highlighted a multifaceted landscape of engagement levels, characterized by both adequate overall engagement and distinct challenges across specific dimensions [51–53]. ISs in Chinese universities generally exhibit adequate overall engagement, surpassing the average level. While their behavioral engagement remains higher, ISs majoring in the humanities show significantly higher levels of behavioral engagement than those majoring in science and technology fields [51]. Nevertheless, some proactive learning behaviors, including low extra-curricular participation, peer interaction, and underutilized institutional support, limit their full integration into academic and socio-cultural engagement [52]. Emotionally, they demonstrate moderate belongingness; however, language barriers and limited interaction with domestic peers hinder the formation of deeper affective connections, resulting in a low level of satisfaction [53]. In particular, students from Asia and Africa face higher acculturation stress due to rejection, identity threat, opportunity deprivation, and self-confidence issues than those from other regions (America/Europe/Oceania) [54]. Cognitively, their level of cognitive engagement is relatively lower and below the overall average due to systematic barriers, such as cross-cultural classroom dynamics, largely lecture-based pedagogical approaches [51,52], Chinese-dominant instruction with limited interactive and student-centered practices, and more relaxed academic expectations [55,56]. Their agentic engagement remains constrained due to centralized curricula and management inefficiencies [14–16]. Studies have also revealed that, compared to individual factors, environmental factors contribute more to ISs’ overall engagement [57]. Teacher support, peer relationships, and campus management support have a significant positive impact on their engagement, with teacher support exerting the most significant influence [58].

Comparative studies indicate that ISs from Western countries tend to achieve better socio-cultural adaptation than those from other regions, and students in integrated programs (where international and domestic studies are combined) tend to adapt more strongly than those in segregated programs [18]. Regarding individual factors, Chinese language proficiency is the most frequently reported predictor, with no significant difference in grades. In particular, ISs from Western countries appear to outperform their Asian and African counterparts in Chinese language proficiency [18,58,59]. However, Tian et al. [18] observed significant variations based on gender and discipline among the ISs at China’s double-first-class universities, with no significant variation across grade levels. Notably, male students were found to be more actively engaged than their female counterparts. Among the disciplines, medical students exhibited the highest level of active engagement, followed by those in the humanities, social sciences, and engineering. Importantly, students at double first-class universities demonstrate greater engagement in learning than those at non-double first-class institutions. Supporting the above-mentioned findings, [19] contends that ISs in Chinese universities actively develop cognitive, affective, and behavioral strategies to overcome language barriers and psychological adjustments; however, these efforts are often inadequate when faced with such structural constraints.

A critical knowledge gap persists in existing literature. First, although SE has been acknowledged as multidimensional, few studies have systematically examined how specific determinants, such as cultural adaptation, self-efficacy, language proficiency, and teacher and peer affect, contribute to each of the core SE dimensions. Second, studies suffer from narrow sampling bias, characterized by the overrepresentation of elite universities and regions (e.g., Beijing and Northeastern China) and particular nationalities (e.g., New Zealand and Korea) [14,16], which limits generalizability to non-elite institutions and less developed provinces, such as Yunnan and Guangxi, each with only one double first-class university. Third, hypothesis-driven analyses examining the causal pathways between predictors and engagement dimensions are scarce, constraining theoretical interpretability.

To address this gap, the present study tests the following three hypotheses:

H1: Engagement levels differ significantly according to gender, academic discipline, and grade.

H2: Emotional engagement exceeds behavioral and cognitive engagement.

H3: Individual factors (e.g., cultural adaptation and self-efficacy), teacher support, and peer support significantly predict SE.

These hypotheses were tested using Structural Equation Modelling (SEM) and supported by scale-based surveys grounded in previously validated instruments.

## 3 Methodology

### 3.1 Ethics and consent

This study was supported by Yunnan Normal University’s 2024 Postgraduate Scientific Research Fund, key project “Influencing Factors and Intervention Research on Chinese Learning Engagement for Undergraduate Students in China,” grant number YJSJJ24-A08.

The study protocol was reviewed and approved by the Academic Committee for Doctoral Studies at Yunnan Normal University, confirming its alignment with institutional ethical guidelines for non-sensitive educational research and ensuring the safeguarding of participants’ rights and well-being.

Participation was explicitly voluntary for both groups. For offline participants, verbal consent was obtained after the first author explained the study’s objectives, its voluntary nature, and the measures in place to ensure anonymity and confidentiality, with no coercion applied. For online participants, a mandatory informed consent checkbox at the start of the *Wenjuanxing* survey outlined these details, and only those who consented could proceed. A small incentive (1–5 RMB) was offered exclusively to online participants. This incentive took the form of an automatic digital red envelope (hongbao) distributed via a lottery-style mechanism (choujiang) upon survey completion, which was disclosed beforehand and disbursed digitally without collecting personal identifiers to preserve anonymity. No identifiable data were collected from either group, and all surveys were completed individually, with anonymity and confidentiality strictly maintained throughout the data collection and incentive distribution process.

### Informed consent statement

Informed consent was obtained from all participants involved in the study.

### 3.2 Instrumentation

The questionnaire was adapted from NSSE-China [23], CCSS [25], and MSLQ [26], using a validated translation and back-translation method. It was slightly modified for the international undergraduate context in Chinese higher education. Specifically, the items for the SE dimensions in the questionnaires were adapted from references [23,25], while the items contributing to the SE construct were adopted from references [23,24,26].

Expert reviews and pilot testing were performed. All responses were rated on a 5-point Likert scale with scores ranging from 1 (“completely disagree”) to 5 (“completely agree”). To ensure cross-linguistic equivalence, three bilingual education experts reviewed the final version for content relevance and clarity of the questionnaire. A pilot study involving 38 ISs was conducted to refine the item wording and confirm administrative feasibility; no major revisions were required.

The final instrument consisted of four sections. While the introduction section explained the purpose of the survey, the demographics section measured variables such as gender, age, academic major, grade, Chinese language proficiency (as measured by the Hanyu Shuiping Kaoshi-HSK level), scholarship status, motivation for learning Chinese, and other background factors. Similarly, the SE domains section measured behavioral engagement (11 items, e.g., participation, persistence), cognitive engagement (8 items, e.g., metacognitive strategies, deep learning), and emotional engagement (6 items, e.g., interest, sense of belonging, satisfaction). Finally, the determinants of the SE section comprise three items across three subscales. These include individual factors (11 items measuring cultural adaptation, language proficiency, motivation, and self-efficacy), as well as teacher and peer factors (9 and 11 items, respectively, assessing their behavioral and emotional support, influence, and pressure). The social/environmental factors (8 items), encompassing access to government incentives, support, and the school’s policy and management system, were also included in the questionnaire; however, they were excluded from this study as they demonstrated no significant impact on these students’ engagement. Table 1 presents the dimensions of SE along with their measurement indicators and examples of question items.

**Table 1:** Student engagement dimensions and items.

Dimensions	Measurement Indicators	Sample question items(example)
Behavioural engagement	Concentration, persistence, regularity, participation	Even if I yawn to sleep, I will try to concentrate. I complete classroom tasks as required.
Cognitive engagement	Cognitive strategies, metacognitive strategies	When I study, I take notes on essential concepts, contents, or viewpoints. I have clear goals and a long-term learning plan.
Emotional engagement	Satisfaction, interest, sense of belongingness	I am pleased with my progress in learning Chinese. I feel like a part of the school.

Table 2 presents the predictors of SE, accompanied by measurement indicators and sample question items.

**Table 2:** Adopted measurement for predictors of student engagement.

Variables	Measurement Indicators	Sample question items(example)
Teacher factors	Behavioural support, emotional support	Teachers provide timely feedback on learning tasks or assignments. Teachers tailor their teaching to accommodate our diverse learning styles and backgrounds.
Peer factors	Behavioural support, emotional support	When I do not participate in Chinese learning activities on time, my classmates remind me to complete the task. When I encounter difficulties, students are willing to help me.
Social/ Environmental factors	Family support, government and school support, policy support	My family supports me in studying in China. Schools organize cultural exchange activities (such as the International Cultural Festival for international students). I am eager to share my experiences of Chinese culture with my family and friends upon my return and encourage them to visit China.

### 3.3 Scale validation and reliability

An Exploratory Factor Analysis (EFA) was performed to assess construct validity using principal axis factoring with varimax rotation, a standard approach for scale validation in psychological and educational measurement [60]. The sampling adequacy and factorability of the data were assessed using the Kaiser-Meyer-Olkin (KMO) measure and Bartlett’s Test of Sphericity [61]. Bartlett’s Test, rooted in foundational factor analysis methodology [61], was used to verify the suitability of the correlation matrix for factor extraction. Factors were retained based on the eigenvalue criterion of greater than 1, and the scree plot method was employed to determine the optimal number of common factors, following established guidelines [60].

Reliability was primarily accessed using Cronbach’s alpha. In addition, inter-item correlations and item-total statistics were also examined during scale refinement to insure internal consistency and construct robustness.

### 3.4 Data collection and sample characteristics

Data were collected between June and September 2024 from 21 non-double-first-class universities in Guangxi and Yunnan via Wenjuanxing, WeChat, QQ, and class settings coordinated with instructors. For the offline surveys, the first author administered paper-based questionnaires during class hours, without the teacher’s presence, to minimize bias. Participants completed paper-based questionnaires individually, and no group discussions were involved. In contrast, online surveys were completed independently by the participants outside school hours via their own personal devices. The inclusion criteria were: (1) international undergraduate status with at least one semester of enrollment, and (2) currently enrolled in Chinese education courses at 21 non-double first-class universities in Guangxi and Yunnan provinces, especially international education schools.

A total of 750 responses were initially collected, with 29 excluded due to incomplete or patterned responses, resulting in 721 valid entries for analysis in SPSS 22.0. This achieved a 100% response rate and a 97.4% validity rate. As shown in Table 3, the sample consisted of 64% females and 36% males. Regarding their age, 24% were under 20, 60% were between 21 and 25, 12% were 26 and 30, and 4% were 31 or older. The majority of students were from liberal arts (72%), followed by medical studies (21%) and science and engineering (7%). Regarding academic standing, the breakdown was as follows: 35% were first-year students, 20% were sophomores, 27% were juniors, and 19% were seniors. Regarding their Chinese language proficiency, 46% had achieved HSK level 4, and 24% had reached HSK level 5. Additionally, 70% of the students were scholarship recipients. Regarding the motivation factors for students to study Chinese, 33% of them were interested in the language, 24% were interested in Chinese culture, 17% needed it for employment, 10% studied it for socializing, 7% for travel purposes, and 4% due to parental influence.

**Table 3:** Characteristics of corpora distribution (n = 721).

Variables	Options	Frequency	Percentage
Gender	M	261	36
	F	460	64
Age	Below 20 years	171	24
	21 ~ 25 years	434	60
	26 ~ 30 years	87	12
	31 years and above	29	4
Specialization	Liberal arts	519	72
	Science and Engineering	53	7
	Medical studies	149	21
Grade	First-year	249	35
	Second-year	145	20
	Third-year	192	27
	Fourth-year	135	19
Learning Chinese duration	Below 1 year	105	15
	1 ~ 3 years	333	46
	4 ~ 6 years	215	30
	6 years above	68	9
Preferred sitting benches during class hours	First-bencher	227	32
	Mid-bencher	380	53
	Back-bencher	114	16
Chinese proficiency level	HSK level 1–3	140	19
	HSK level 4	328	46
	HSK level 5	171	24
	HSK level 6	52	7
	Others	30	4
Scholarship student or not	Yes	506	70
	No	215	30
Reasons for learning Chinese (Multiple choices)	Interested in the Chinese language	514	33
	Interested in Chinese culture	372	24
	Parents demand	80	5
	Need for social interactions	148	10
	Professional need	269	17
	For tourism	114	7
	Others	59	4

### 3.5 Statistical analysis

All data analyses were conducted in sequential stages to ensure analytical rigor and consistency. Descriptive statistics (means and standard deviations) were first computed for all study variables, followed by independent-sample t-tests and one-way ANOVA to examine group differences across demographic variables, including gender, discipline, and grade level. Effect sizes were calculated using Cohen’s d for t-tests following Cohen’s benchmarks(d = 0.20 = small; d = 0.50 = medium; d = 0.80 = large) [62], and partial eta squared (η²)for ANOVA.

Pearson correlation and multiple regression analysis were subsequently performed to examine associations among engagement dimensions and the relative contributions of individual factors and contextual factors. Prior to regression analysis, standard statistical assumptions were tested, including normality (via the Shapiro–Wilk test), homogeneity of variances (using Levene’s test), and absence of multicollinearity (through variance inflation factor (VIF) calculations), following established recommendations in statistical learning [60].

Building on these preliminary analyses, a Confirmatory Factor Analysis (CFA) was performed using maximum likelihood estimation in AMOS 24.0 to validate the measurement model and assess construct reliability and validity. Model fit was evaluated using χ²/df, Comparative Fit Index (CFI), Tucker-Lewis Index (TLI), Root Mean Square Error of Approximation (RMSEA), and Standardized Root Mean Square Residual (SRMR) following the recommended threshold of Hu and Bentler [62].

Structural Equation Modeling (SEM) was conducted to test the hypothesized relationship. Student engagement was specified as a latent construct represented by behavioral, cognitive, and emotional dimensions. Consistent with regression results, the model incorporated the most influential predictors: cultural adaptation and self-efficacy (individual factors), as well as teacher behavioral support and peer behavioral support (contextual factors). Chinese proficiency and emotional support factors were excluded due to their non-significant or minimal impact in earlier analyses. SEM model was assessed using the same indices to ensure consistency with the measurement model. Standardized path coefficients(β), significance levels(p values), and explained variance(R²) were reported to evaluate the hypothesized relationship.

## 4 Results

### 4.1 Psychometric analysis of student engagement

As presented in Table 4, findings from EFA conformed strongly to sampling adequacy and factorability, supported by the KMO measure and Bartlett’s Test of Sphericity. For the SE scale, the KMO measure of sampling adequacy was 0.94, indicating excellent sampling adequacy [60]. Bartlett’s sphericity test was highly significant (χ² (276)=3456.78, p < 0.001), and the cumulative variance contribution rate was 67.89%. These results confirm that the correlation matrix was suitable for factor extraction and that the constructs demonstrate strong validity [61].

**Table 4:** Validity analysis of SE and predictors.

Measure	KMO	Degree of freedom	Statistical significance	Cumulative variance contribution rate
SE dimensions	0.94	276	0.000	67.89
Predictors of SE	0.96	666	0.000	70.77

Similarly, predicators to SE showed an excellent KMO value of 0.96, with a significant Bartlett’s sphericity test (χ² (666) =7890.34, p < 0.001), with a cumulative variance contribution rate of 70.77%, meeting standard benchmarks for acceptable validity, supporting the suitability of the data for factor analysis [60]. Additionally, the scree plot method confirmed the extraction of 1–3 common factors per dimension, collectively indicating strong construct validity for both the SE scale and its predictors.

As shown in Table 5, all SE dimensions and contributing factors demonstrated high internal consistency (α > 0.9), indicating excellent internal consistency exceeding the 0.7 threshold for acceptable reliability. Supplementary checks of the inter-item correlations further confirmed the robustness of these measures.

**Table 5:** Reliability analysis of SE and predictors.

Variables	Cronbach’s Alpha (α)	Number of Items
Behavioural engagement	0.923	11
Cognitive engagement	0.905	8
Emotional engagement	0.903	6
Overall engagement	0.951	25
Teachers support	0.958	9
Peer support	0.954	11
Personal factors	0.949	11
Predictors of SE	0.973	31

Following the exploratory factor analysis, a confirmatory factor analysis (CFA) was conducted to further verify the factorial validity and measurement quality of the constructs before testing the structural model.

### 4.2 Confirmatory factor analysis

A confirmatory factor analysis(CFA) conducted in AMOS 24.0 demonstrated excellent measurement model fit: χ² (180) =395.28, χ²/df = 2.20, CFI = 0.98, TLI = 0.97,RMSEA = 0.04(90% CI = 0.03–0.05), and SRMR = 0.03. Standardized factor loadings ranged from 0.70 to 0.78 (all p < 0.001). Composite reliability (CR) values ranged from 0.86 to 0.93, and average variance extracted (AVE) values ranged from 0.58 to 0.72, indicating convergent validity.

### 4.3 Descriptive analysis of student engagement

One of the primary aspects of SE lies in understanding the factors that help students engage and intervene to get them back on track when they become disengaged from their studies. Table 6 shows that among 721 international undergraduates, the overall mean score was 4.02 (SD = 0.63), indicating a medium to high level of engagement. Emotional engagement scored the highest (M = 4.10, SD = 0.72), followed by behavioral (M = 3.99, SD = 0.66) and cognitive engagement (M = 3.98, SD = 0.72). These findings suggest that students feel a stronger affective connection to their academic experience than they exhibit in their observable behaviors or cognitive strategies.

**Table 6:** Descriptive statistics for engagement dimensions (N = 721).

Variables	N	Minimum (Min)	Maximum (Max)	Mean	Standard Deviation (SD)	Interpretation
Behavioural engagement	721	1	5	3.99	0.66	Moderate
Cognitive engagement	721	1	5	3.98	0.72	Moderate
Emotional engagement	721	1	5	4.1	0.72	Moderately high
Overall engagement	721	1	5	4.02	0.63	Moderate

**Note:** Emotional engagement emerged as the most prominent dimension.

### 4.4 Group differences in student engagement

#### 4.4.1 Gender-based differences.

Independent sample t-tests were conducted to examine gender differences in the dimensions of engagement. The independent sample t-tests (see Table 7) revealed that male students scored higher than their female counterparts across all engagement dimensions. Statistically significant differences were found for cognitive engagement (t = 3.45, p = 0.001, Cohen’s d = 0.27) and overall engagement (t = 2.162, p = 0.031, Cohen’s d = 0.17). In contrast, the differences for behavioral engagement (t = 1.915, p = 0.056, Cohen’s d = 0.15) and emotional engagement (t = 0.547, p = 0.585, Cohen’s d = 0.04) were not statistically significant. According to Cohen’s (1988) benchmarks, where d = 0.2 represents a small effect, d = 0.5 a medium effect, and d = 0.8 a large effect, all the observed effects in this analysis fall within the small range. This indicates that while gender differences, particularly in cognitive engagement, are statistically reliable, the magnitude of these differences is modest in practical terms.

**Table 7:** Gender differences in engagement.

Variables	Gender	N	Mean	SD	SEM*	t	SS	Effect Size (Cohen’s d)	Interpretation
Behavioural engagement	M	261	4.057	0.712	0.044	1.915	0.056	0.15	ns
	F	460	3.959	0.629	0.029				
Cognitive engagement	M	261	4.102	0.750	0.046	3.45	0.001	0.27	
	F	460	3.912	0.687	0.032				
Emotional engagement	M	261	4.119	0.785	0.049	0.547	0.585	0.04	ns
	F	460	4.089	0.686	0.032				
Overall engagement	M	261	4.087	0.676	0.042	2.162	0.031	0.17	
	F	460	3.982	0.597	0.028				

* SEM = Standard Error of Mean; SS = Statistical Significance; ns = not significant.

#### 4.4.2 Differences across academic disciplines.

A one-way ANOVA revealed significant differences in engagement across academic disciplines. As presented in Table 8, variations in SE across academic disciplines were statistically significant for behavioral engagement (F = 9.807, p < 0.001, η² = 0.027), cognitive engagement (F = 10.25, p < 0.001,η² = 0.028), and overall engagement (F = 7.621, p = 0.001, η² = 0.021). In contrast, emotional engagement did not differ significantly (F = 1.129, p = 0.324,η² = 0.003). Subsequent analysis showed that students from medical studies recorded the highest mean scores across most dimensions. Post-hoc comparisons using Tukey’s Honestly Significant Difference (HSD) test confirmed significant disparities between the liberal arts and medical majors. The η² value indicate small- to -moderate practical significance, suggesting that while the observed differences are statistically robust, their real-world impact is modest.

**Table 8:** Variations in academic disciplines.

Variables	Specialization	N	Mean	SD	SEM	F	SS	Effect Size(η²)
Behavioural engagement	Liberal arts	519	3.933	0.641	0.028	9.807		.027
	Science and Engineering	53	4.013	0.700	0.096			
	Medical studies	149	4.201	0.678	0.056			
Cognitive engagement	Liberal arts	519	3.906	0.672	0.030	10.25		.028
	Science and Engineering	53	4.142	0.680	0.093			
	Medical studies	149	4.181	0.824	0.067			
Emotional engagement	Liberal arts	519	4.075	0.673	0.030	1.129	0.324	.003
	Science and Engineering	53	4.148	0.749	0.103			
	Medical studies	149	4.170	0.869	0.071			
Overall engagement	Liberal arts	519	3.966	0.584	0.026	7.621	0.001	.021
	Science and Engineering	53	4.088	0.653	0.090			
	Medical studies	149	4.187	0.735	0.060			

#### 4.4.3 Grade-based differences.

The results in Table 9 indicate that significant variation was found only in cognitive engagement across grades (F = 4.015, p = 0.008, η² = 0.017), with first- and second-year students scoring higher than third- and fourth-year students. No significant differences were observed in behavioral (F = 2.485,p = 0.060,η² = .010), emotional (F = 0.767,p = 0.513,η² = .003), or overall engagement (F = 2.569,p = 0.053,η² = .011). These findings may reflect possible disengagement or a decline in cognitive effort in later academic years, possibly due to academic fatigue or a reduced level of challenge for students.

**Table 9:** Grade-based variations.

Variables	Grade	N	Mean	SD	SEM	F	SS	η²
Behavioural engagement	First-year	249	4.050	0.614	0.039	2.485	0.06	.010
	Second-year	145	4.064	0.712	0.059			
	Third-year	192	3.917	0.583	0.042			
	Fourth-year	135	3.926	0.772	0.066			
Cognitive engagement	First-year	249	4.057	0.679	0.043	4.015	0.008	.017
	Second-year	145	4.064	0.781	0.065			
	Third-year	192	3.848	0.694	0.050			
	Fourth-year	135	3.937	0.716	0.062			
Emotional engagement	First-year	249	4.135	0.707	0.045	0.767	0.513	.003
	Second-year	145	4.117	0.837	0.070			
	Third-year	192	4.034	0.705	0.051			
	Fourth-year	135	4.110	0.645	0.056			
Overall engagement	First-year	249	4.076	0.593	0.038	2.569	0.053	.011
	Second-year	145	4.079	0.716	0.059			
	Third-year	192	3.931	0.577	0.042			
	Fourth-year	135	3.982	0.651	0.056			

#### 4.4.4 Chinese language proficiency.

As shown in Table 10, there were no statistically significant differences across HSK levels(all p > .05) in behavioral (F = 0.653, p = 0.625,η² = 0.002), cognitive (F = 1.289, p = 0.273,η² = 0.004), emotional (F = 2.307, p = 0.057, η² = 0.008), or overall engagement (F = 1.027,p = 0.392,η² = 0.003). The η² values are negligible, indicating that language proficiency exerted minimal practical influence on engagement outcomes. Contrary to expectations, students with higher HSK scores did not demonstrate significantly greater engagement, suggesting that language proficiency alone may not be a key predictor of SE, possibly due to compensatory institutional or teacher, peer support mechanisms.

**Table 10:** Chinese language proficiency-based variations.

Variables	Chinese proficiency	N	Mean	SD	SEM	F	SS	η²
Behavioural engagement	HSK level 1–3	140	4.005	0.728	0.062	0.653	0.625	.002
	HSK level 4	328	3.971	0.657	0.036			
	HSK level 5	171	4.057	0.602	0.046			
	HSK level 6	52	3.923	0.629	0.087			
	Others	30	3.967	0.765	0.140			
Cognitive engagement	HSK level 1–3	140	4.049	0.676	0.057	1.289	0.273	.004
	HSK level 4	328	3.954	0.765	0.042			
	HSK level 5	171	4.031	0.634	0.048			
	HSK level 6	52	3.827	0.678	0.094			
	Others	30	3.928	0.818	0.149			
Emotional engagement	HSK level 1–3	140	4.174	0.670	0.057	2.307	0.057	.008
	HSK level 4	328	4.021	0.805	0.044			
	HSK level 5	171	4.170	0.609	0.047			
	HSK level 6	52	4.228	0.570	0.079			
	Others	30	4.000	0.782	0.143			
Overall engagement	HSK level 1–3	140	4.066	0.617	0.052	1.027	0.392	.003
	HSK level 4	328	3.980	0.674	0.037			
	HSK Level 5	171	4.082	0.545	0.042			
	HSK Level 6	52	3.983	0.543	0.075			
	Others	30	3.965	0.737	0.135			

### 4.5 Correlational analysis of student engagement

Pearson’s correlation analysis (Table 11) revealed strong, positive relationships between the three engagement dimensions and overall engagement (all p < .001). The strongest correlations were between behavioral and overall engagement (r = 0.921) and cognitive and overall engagement (r = 0.909). Emotional engagement was also strongly correlated with overall engagement (r = 0.878). These strong intercorrelations suggest a conceptual overlap among the SE dimensions, although they remain theoretically distinct from each other.

**Table 11:** Correlational analysis of SE dimensions.

Variables	Behavioural engagement	Cognitive engagement	Emotional engagement		Learning engagement
Behavioural engagement	1				
Cognitive engagement	.753**	1			
Emotionalengagement	.684**	.741**	1		
Overall engagement	.921**	.909**	.878**		1

Note: **Correlation is significant at the 0.01 level (two-tailed).***

### 4.6 Regression analysis of student engagement predictors

#### 4.6.1 Individual factors.

Multiple regression analysis was conducted with cultural adaptation, self-efficacy, language proficiency, and learning motivation as predictors of SE. Table 12 shows that cultural adaptation (β = .35, p < .001) (β = .35, p= < .001, 51.9% variance explained) had the most significant effect on SE, followed by self-efficacy (β = .34, p=<.001, 8.2%), Chinese proficiency (β = .12, p = .005), and learning motivation (β = 0.069, 0.2%). The model’s R² was 0.608, indicating that the four predictors collectively accounted for 60.8% of the variance in the SE. These results highlight that cultural adaptation and self-efficacy together explain over 50% of the total variance in SE, providing strong evidence of their practical importance in understanding and influencing student engagement.

**Table 12:** Multiple regression analysis (Individual factors → SE).

	Unstandardized coefficient (B)	standardized coefficient (Beta)	Correlation coefficient (R)	R-squared (R²)	Adjusted R-squared	SS	Tolerance	VIF
Constant	1.027					0.000		
Cultural adaptation	0.313	0.35	0.72	0.519	0.519	0.000	0.312	3.2
Self-efficacy	0.264	0.336	0.775	0.601	0.6	0.000	0.391	2.559
Chinese proficiency	0.092	0.115	0.779	0.606	0.605	0.005	0.338	2.96
Learning motivation	0.054	0.069	0.78	0.608	0.606	0.008	0.421	2.377

#### 4.6.2 Teachers’ and peer support.

Table 13 reveals that teacher behavioral support had a much more substantial impact on SE (β = 0.588, explaining 55% of the variance) than emotional support (β = 0.178, explaining 0.8% of the variance). Collectively, these two variables had a strong association with SE (R = 0.747), accounting for 55.8% of the total variance (R² = 0.558).

**Table 13:** Multiple regression analysis of teacher support.

	Unstandardized coefficient (B)	Standardized coefficient (Beta)	Correlation coefficient (R)	R-squared (R²)	Adjusted R-squared	Statical significance	Tolerance	VIF
Constant	1.431					0.000		
Behavioural support	0.496	0.588	0.742	0.55	0.549	0.000	0.254	3.935
Emotional support	0.142	0.178	0.747	0.558	0.557	0.000	0.254	3.935

As presented in Table 14, peer behavioral support exerted a substantially greater effect on SE (β = 0.493), uniquely explaining 45.9% of the variance, compared with peer emotional support (β = 0.22), which explained 1.5% of the variance. Collectively, these peer support factors had a strong association with SE (R = 0.688), accounting for 47.4% of the variance (R² = 0.474). Additionally, emotional support contributed marginally (≤ 1.5%) to both models, suggesting that while socio-emotional encouragement may have a supplementary role, behavioral support and strategies such as clear guidance, constructive feedback, and collaborative peer interactions, rather than emotional encouragement, are the primary institutional drivers of SE.

**Table 14:** Multiple regression analysis of peer support.

	Unstandardized coefficient (B)	Standardized coefficient (Beta)	Correlation coefficient (R)	R-squared (R²)	Adjusted R-squared	Statical significance	Tolerance	VIF
Constant	1.88					0.000		
Behavioural support	0.38	0.493	0.678	0.459	0.458	0.000	0.296	3.382
Emotional support	0.168	0.22	0.688	0.474	0.472	0.000	0.296	3.382

### 4.7 Structural Equation Modeling (SEM) analysis

Building on the validated measurement model from the CFA (section 4.2), the SEM model was estimated in AMOS 24.0 using maximum likelihood estimation. The SEM demonstrated an excellent fit, consistent with the CFA results, confirming that the hypothesized structural relationships adequately represented the observed data (χ²/df = 2.12, CFI = 0.98, TLI = 0.97, RMSEA = 0.04, SRMR = 0.03).

**Fig 1:**
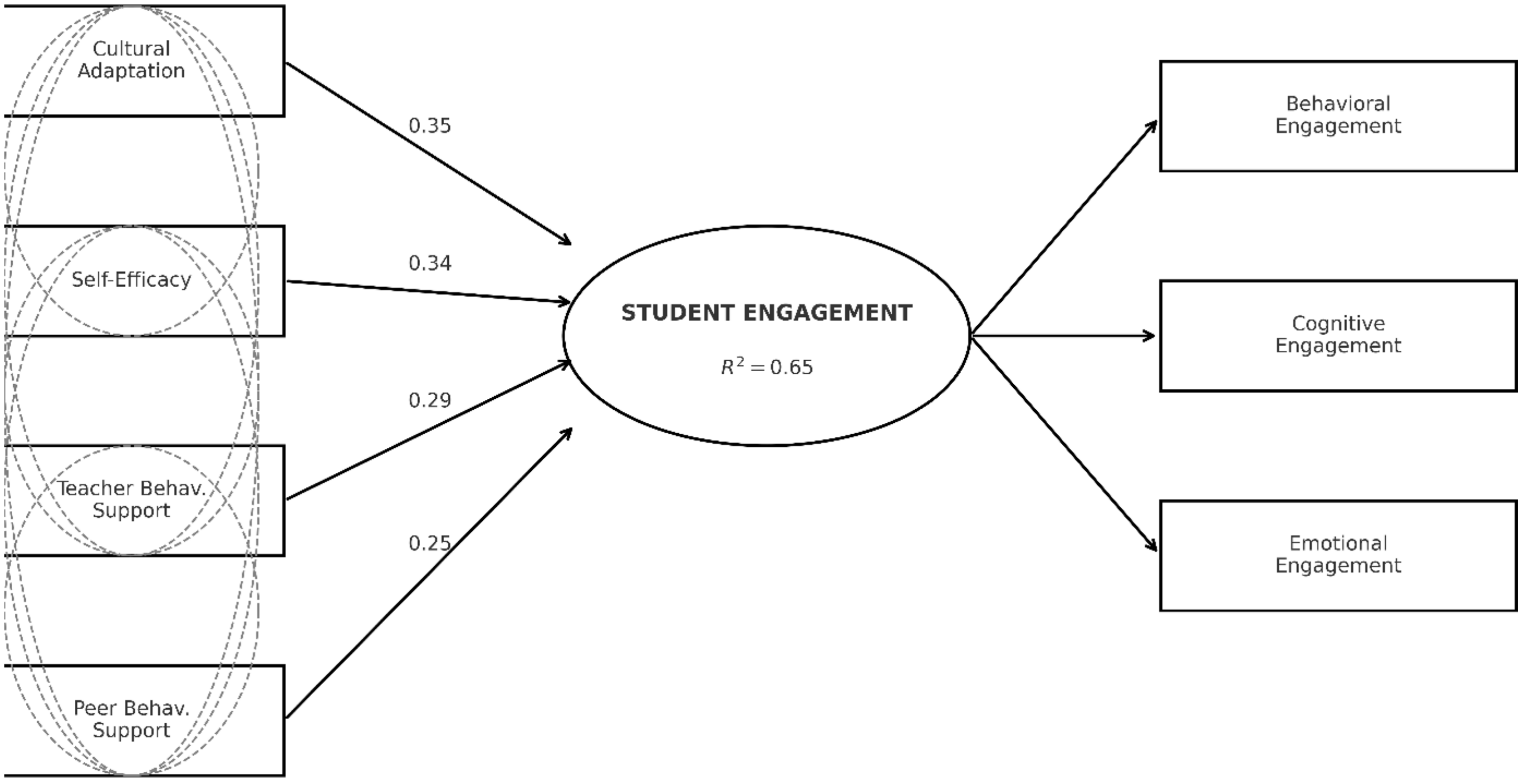
SEM path model of student engagement.

The results of the path analysis are presented in Table 15 and illustrated in Fig 1. All four hypothesized paths were statistically significant. Cultural adaptation (β = 0.35, p < .001) was the strongest predictor, followed closely by Self-Efficacy (β = 0.34, p < .001). Teacher Behavioral Support (β = 0.29, p < .001) and Peer Behavioral Support (β = 0.25, p < .001) were also strong, significant predictors. Collectively, these four factors explained a substantial 65% of the variance in overall Student Engagement (R² = 0.65). These SEM results provide robust support for H3, confirming that individual factors and behavioral support from teachers and peers are significant predictors of international student engagement.

**Table 15:** Structural Equation Modeling (SEM) path coefficients and model fit indices.

Hypothesized Path	Standardized Estimate (β)	p-value	Support for H3
Cultural adaptation → Student Engagement	0.35	<.001	Yes
Self-Efficacy → Student Engagement	0.34	<.001	Yes
Teacher Behavioral Support → Engagement	0.29	<.001	Yes
Peer Behavioral Support → Engagement	0.25	<.001	Yes

In summary, the above findings revealed a medium-high level of overall engagement among international undergraduates, with emotional engagement being the most prominent dimension. Significant gender differences were observed in cognitive and overall engagement, with males scoring higher. Engagement varied substantially across disciplines, particularly in cognitive engagement, with medical students demonstrating the highest levels of engagement. Similarly, while cognitive engagement declined in higher grades (third and fourth years), it partially supported group differences (H1). Conversely, language proficiency (HSK-Level) was not significantly associated with engagement.

Regarding the SE dimensions, emotional engagement exhibited the highest mean score, exceeding that of behavioral and cognitive engagement. Further supporting H2, all engagement dimensions were strongly intercorrelated, particularly behavioral and cognitive engagement with overall engagement, confirming a dimensional overlap (Table 11). Despite the high intercorrelations among SE dimensions, each dimension contributes uniquely to overall engagement, highlighting the importance of fostering adaptive, self-efficacious learning environments while prioritizing behavioral support mechanisms to enhance the engagement of international undergraduate students across various educational contexts.

Regression analysis and Structural Equation Modeling (SEM) identified individual factors (cultural adaptation, self-efficacy) and behavioral support from both teachers and peers as the most impactful predictors of SE. However, emotional support from both sources had more minor but significant effects; practical and actionable support mechanisms proved to be substantially more impactful. The SEM model (H3) demonstrated an acceptable fit, robustly conforming to the critical role of structured support systems and individual resources in enhancing engagement among international undergraduate students in Chinese universities.

## 5 Discussion

This study examined the engagement levels of 721 international undergraduates in 21 Chinese non-double-first-class universities in Yunnan and Guangxi provinces, using a multidimensional approach that spanned behavioral, cognitive, and emotional lenses, and tested three hypotheses. It further explored demographic and academic group differences and assessed the predictive strengths of individual, teacher, and peer factors. The results support H1, indicating that significant group differences emerged by gender, academic major, and year of study. H2 is also supported, as emotional engagement had the highest score. H3 is supported by SEM results, which show that individual, teacher, and peer factors significantly predict student engagement. The findings provide empirical insights into how engagement manifests in China’s international education landscape and offer guidance for institutional improvements.

### 5.1 Interpretation of key findings

#### 5.1.1 Emotional engagement was the highest-scoring dimension.

The prominence of emotional engagement (M = 4.10) suggests that ISs maintain a strong affective connection to their learning environment. This finding aligns with previous research indicating that emotional engagement often reflects students’ sense of belonging, satisfaction, and perceived support [1,2]. It also implies that students may feel welcomed and emotionally supported despite challenges in behavioral or cognitive participation [51].

However, the relatively lower cognitive engagement (M = 3.98) raises concern. Cognitive engagement involves metacognitive strategies, deep learning, and sustained mental effort [3]. The finding suggests that instructional practices may not be sufficiently effective in promoting higher-order thinking [18]. This echoes critiques of Chinese higher education’s traditional reliance on teacher-centered, lecture-based instruction [11,12,14,15], which may limit students’ active construction of knowledge. The gap between emotional and cognitive engagement highlights an opportunity for pedagogical reform—specifically, the adoption of more inquiry-based, collaborative, and student-centered learning strategies.

#### 5.1.2 Although small, gender differences were statistically and practically significant.

Gender effects may reflect socio-cultural roles or educational exposure, with men reporting higher cognitive and overall engagement than women (Cohen’s d = 0.27 and 0.17, respectively). These findings are consistent with prior work that has noted gendered patterns in classroom interaction and academic self-concept among ISs, even in double-first-class Chinese universities [18], especially in STEM contexts [4]. While emotional and behavioral engagement showed no significant gender differences, the higher cognitive engagement among men may reflect their greater confidence in problem-solving or a more substantial interest in analytical tasks [38]. However, the effect sizes suggest that interventions to address this disparity, such as gender-responsive classroom practices, may help bridge the gap without requiring structural overhauls.

#### 5.1.3 Discipline and grade level matter.

Students majoring in medicine and engineering exhibited higher behavioral and cognitive engagement than those majoring in liberal arts, supporting the idea that engagement is discipline-specific. STEM fields often require active learning, technical skill acquisition, and problem-solving, naturally encouraging deeper engagement [63]. Conversely, disciplines such as the arts and humanities may cultivate distinct forms of engagement, which are often more attuned to emotional or behavioral aspects. A study by Tian and colleagues [18] on ISs’ engagement also shows that medical students had the highest level of active engagement, surpassing those in humanities, social sciences, and Science and engineering. Notably, despite considerable variations in specific dimensions, the lack of substantial changes in overall engagement suggests that while various forms of engagement differ by discipline, the overall level of engagement remains relatively consistent. It may result from students offsetting diminished cognitive engagement with increased behavioural or emotional engagement in specific domains [51,63].

Similarly, lower-grade students (first- and second-year) reported higher cognitive engagement than upper-grade students. This aligns with the research by Pintrich and De Groot [64], who discovered that students at an advanced academic level (older age cohorts) typically employ more sophisticated learning strategies, reflecting greater cognitive engagement than do their younger counterparts. This may also reflect an early motivation boost upon arrival in China, followed by a decline due to academic fatigue, unmet expectations, or a lack of progressive instructional design [8]. These findings suggest that institutions should target upper-year cohorts with re-engagement strategies such as capstone projects or research-led learning to sustain motivation and effort.

#### 5.1.4 Chinese proficiency was not a significant differentiator.

Contrary to expectations, no significant differences in engagement were observed between the HSK levels. While prior studies have linked language proficiency to academic success and satisfaction with participation [14,65], emphasizing that difficulties with conversing in the host language can lead not only to challenges in learning engagement but also result in considerable stress and a negative self-concept [65]. However, this result may indicate the effectiveness of compensatory mechanisms, such as translation assistance, bilingual teaching, or simplified instructional materials, that help bridge language gaps. Alternatively, this may suggest that engagement is more strongly mediated by institutional and relational support than by language skills alone. This emphasizes the importance of focusing on learning environments rather than language ability when supporting international students.

#### 5.1.5 Cultural adaptation and self-efficacy were the strongest individual predictors.

Together, cultural adaptation and self-efficacy explained over 50% of the variance in SE. This finding reinforces Bandura’s social cognitive theory, which posits that beliefs about one’s capabilities directly influence motivation and persistence. Students who feel integrated into the host culture and are confident in their abilities are more likely to engage meaningfully [30]. This finding echoes the observation that successful cultural adaptation facilitates ISs’ study commitment in China, which can influence the purpose of study (learning goal-oriented) and self-efficacy, as well as language proficiency [36,42,51]. Institutions should thus provide more focus on both psychological (self-efficacy) and socio-cultural (adaptation) dimensions through structured cultural orientation, mentorship programs, and resilience training to optimize their successful transitions [42,51].

#### 5.1.6 Behavioral support mechanisms from teachers and peers outperform emotional support.

Behavioral support (e.g., providing academic guidance, structuring tasks, and organizing collaborative activities) was the most significant institutional predictor of SE. These findings align with Bandura’s assertion that learning is socially mediated through modeled actions, structured guidance, and collaborative academic problem-solving [30]. Prior research highlights the effectiveness of teacher-student interactions, structured feedback, academic scaffolding, and peer-led activities (e.g., group study sessions and academic mentoring) in promoting higher levels of engagement [41–43]. In contrast, while emotional support matters (i.e., improves general well-being), and its impact on active engagement is lower, this underscores that while emotional affirmation is appreciated, actionable support that hinges more on structured interaction (e.g., feedback, scaffolding, goal-setting) is more effective in enhancing academic behaviors and motivation [45]. These results have practical implications: institutions should create structured, culturally adaptive environments and invest in capacity building for teachers, as well as in peer mentoring programs.

#### 5.1.7 High intercorrelations among the SE dimensions require thoughtful interpretation.

Correlations exceeding 0.70 suggest overlapping constructs but do not imply redundancy. Theoretically, these dimensions remain distinct in focus: cognitive engagement targets mental effort, behavioral engagement concerns participation, and emotional engagement reflects affective states [4]. However, their alignment in this study may reflect a contextual “halo effect” where students who feel supported are generally engaged across all fronts. Future research should investigate the interaction effects among these dimensions and consider employing latent variable modeling, such as structural equation modeling (SEM), to isolate the underlying constructs.

### 5.2 Policy and practice implications

These findings underscore the necessity for higher education in China to adopt policies that institutionalize culturally inclusive curricula, faculty training in responsive pedagogy, and peer mentoring programs to enhance equity and student success. At the policy level, universities should mandate ongoing professional development in culturally responsive pedagogy and active learning methods, particularly in lecture-heavy disciplines, to validate students’ diverse backgrounds and provide opportunities for intercultural dialogue. Additionally, introducing structured peer mentoring initiatives, especially for upper-year or academically at-risk students, should be considered, as these programs have been shown to improve retention and academic performance. From a practical standpoint, faculty should integrate early and constructive feedback mechanisms, scaffolded goal-setting exercises, and applied learning opportunities, such as community-based and multidisciplinary projects, to sustain student engagement.

### 5.3 Limitations and future directions

Although the current study provides valuable insights, several methodological and conceptual gaps remain that warrant further investigation. First, the sample is geographically concentrated in non-double-first-class universities across Yunnan and Guangxi provinces. The sample’s imbalance, such as a gender imbalance (64% women) and a concentration in liberal arts (72%), may limit its generalizability to other disciplines or more gender-balanced populations. Expanding the geographical scope to include multiple provinces and diversifying institutional samples would enhance the external validity of the findings and enable meaningful cross-contextual comparisons of the results. Second, the cross-sectional design did not allow for causal inferences. To advance the understanding of ISs’ engagement and its implications, future studies should adopt longitudinal or mixed-methods approaches to capture engagement dynamics over time (across academic stages). Finally, the use of self-report surveys introduces potential biases, including social desirability and recall biases. Subsequent studies should investigate the relationship between engagement and academic performance, well-being, and retention outcomes to clarify the long-term impacts.

Furthermore, with the emergence of remote learning and technological advancements, the growing employment of AI in classroom and assessment activities has been shifting SE priorities, ultimately leading to improved academic performance and student satisfaction, and facilitating the development of tailored support systems in higher education. Future studies should focus on utilizing AI to optimize ISs’ engagement and learning outcomes. These methodological and contextual refinements will strengthen the empirical foundation for evidence-based interventions in higher education.

## 6 Conclusion

This study investigated the engagement of international undergraduate students across three dimensions—behavioral, cognitive, and emotional—alongside the predictors of these dimensions within Chinese higher educational institutions in Yunnan and Guangxi provinces. By addressing three core research questions, it provides several important conclusions. First, international undergraduates in China demonstrate a moderate to high overall engagement level, with emotional engagement emerging as the most prominent dimension. This indicates that students experience a sense of belonging and affective satisfaction despite fluctuations in their behavioral and cognitive effort. (2) Second, modest yet significant demographic differences were identified, especially male students showed higher cognitive and overall engagement; students in medical and STEM disciplines scored higher across dimensions than those in liberal arts; and first- and second-year students reported greater cognitive engagement than upper-year peers. Notably, no significant differences were observed across Chinese proficiency levels, challenging the presumption that language is a primary barrier to ISs’ engagement. Third, cultural adaptation, self-efficacy, and behavioral support from both teachers and peers were the most influential predictors, collectively accounting for over 50% of the variance in SE and affirming their critical role in supporting the success of ISs in China’s higher education.

In summary, this study provides empirical evidence that both individual characteristics and behavioral support within the academic learning environment shape the engagement of international undergraduate students in China. These findings underscore the importance of institutions adopting a proactive, multidimensional approach to create inclusive, engaging, and empowering learning environments for international students.
